# Pelvic Endoprosthesis after Hemipelvectomy Using a 3D-Printed Osteotomy Guide for Infiltrative Osteoma in a Cat

**DOI:** 10.3390/vetsci9050237

**Published:** 2022-05-16

**Authors:** Yoonho Roh, Jaemin Jeong, Youngjin Jeon, Daehyun Kim, Seongmok Jeong, Haebeom Lee

**Affiliations:** College of Veterinary Medicine, Chungnam National University, Daejeon 34134, Korea; royoonseok@gmail.com (Y.R.); klmie800@cnu.ac.kr (J.J.); orangee0115@ogmail.com (Y.J.); vet1982@cnu.ac.kr (D.K.); jsmok@cnu.ac.kr (S.J.)

**Keywords:** 3D printing, cat, hemipelvectomy, osteoma

## Abstract

With the development of 3D printing and surgical techniques, various defect reconstruction methods after tumor resection have been applied not only in humans but also in veterinary medicine. This report describes a case of reconstruction after hemipelvectomy for an osteoma in a cat using a 3D-printed pelvic endoprosthesis and micro total hip replacement (mTHR). A 5-year-old spayed female Turkish Angora cat was referred for a 1-month history of constipation and intermittent weight-bearing lameness in the left hindlimb. An osteoma in the pelvis measuring 4.5 × 3 × 5.4 cm was identified based on diagnostic examinations. A left mid-to-caudal partial and right caudal partial hemipelvectomy, and a left femoral head and neck osteotomy, were planned to remove the mass. Reconstruction of the bone defect using 3D-printed metal endoprosthesis and mTHR in the left hindlimb was intended. During right caudal partial hemipelvectomy, right femoral head and neck osteotomy was performed because there was infiltration in the medial wall of the acetabulum. Histopathological examination confirmed the diagnosis of an osteoma. Two weeks post-surgery, surgical debridement and femoral stem removal were performed because of delayed wound healing and sciatic neurapraxia, leading to femoral stem dislocation from the cup. The delayed wound healing and sciatic neurapraxia were appropriately addressed. The cat regained normal weight and defecation 4 weeks post-operatively. Two years post-surgery, the patient recovered with an almost normal gait. Hemipelvectomy with 3D-printed endoprosthesis provides a safe surgical option with favorable outcomes for neoplasms in the pelvis of cats.

## 1. Introduction

Primary bone tumors, including osteoma, osteosarcoma, chondrosarcoma, multiple myeloma, and fibrosarcoma, are less commonly reported in cats [1,2]. Osteomas are protruding tumor masses and benign neoplasms composed of histologically abnormally dense but otherwise normal bone formed in the periosteum [3]. Osteoma is a continuously slow-growing mass and generally does not cause clinical signs unless it interferes with adjacent structures [1,4]. Osteomas are rare in cats and arise mainly from the flat bones of the head [1,4]. Tumors of the skeleton require a multimodal approach for treatment, such as surgery, radiation, and chemotherapy. Limb amputation of the appendicular skeleton and aggressive resection of the axial skeleton are the principal treatments for this type of tumor [5]. Resection of the pelvic segment (hemipelvectomy) is an aggressive but general surgery to remove a pelvic segment for the treatment of tumors, trauma, and malunion in the pelvis [6,7]. In dogs and cats, hemipelvectomy is a salvage treatment categorized as total, mid-to-caudal partial, mid-to-cranial partial, and caudal partial [6]. The extent of hemipelvectomy is determined based on the type of tumor and the aim of the surgery, such as corrective surgery for malunion [4]. Internal hemipelvectomy preserves limb function if the tumor does not invade the major neurovascular structures [8].

Although it is reported that post-operative complications after hemipelvectomy in dogs and cats were uncommon and usually minor, hemipelvectomy is still a difficult and extensive procedure. Amputation of the ipsilateral hindlimb for complete resection of the pelvic tumor requires consideration of the regional anatomic circumstance of the patient with regard to the orthopedic, neurologic, and oncologic aspects [6,9,10,11,12]. A broad surgical margin should be obtained to ensure complete tumor removal for better survival and reduced recurrence [6]. By properly securing the surgical margin, tumor recurrence can be reduced; however, the weight-bearing function can be lost [13,14]. Resection of the pelvic tumor and reconstruction of the remaining defect is important to restore the locomotive motion function. Load transfer from the axial skeleton to the lower extremity could be sustained [10,15,16,17].

With the advancements in surgical techniques and various reconstruction methods for defects after bone removal, the feasible application of an endoprosthetic reconstruction in humans has been recently reported with favorable outcomes in terms of stable fixation, early mobilization, and osteointegration [13,15]. Additionally, endoprosthetic reconstruction can achieve more functional restoration than simple hemipelvectomy with femoral head and neck osteotomy (FHNO) [10,18]. Recently, with the help of 3D printing, patient-specific endoprostheses have been developed and manufactured for accurate alignment and the immediate restoration of function and stability [15,17]. In veterinary medicine, endoprosthesis placement for limb-sparing procedures has been reported for allografts, metals, and orthopedic implants [12,16]. Although a previous report in a dog described a successful limb salvage with an endoprosthesis that replaced the acetabulum and total hip replacement (THR), limited information is published on cats regarding hemipelvectomy with endoprosthesis [10].

This report describes a 3D-printed resection guide and pelvic endoprosthesis for hemipelvectomy as a viable surgical method in an osteoma of a cat, with a good long-term outcome.

## 2. Case Description

### 2.1. Case

**Figure 1:**
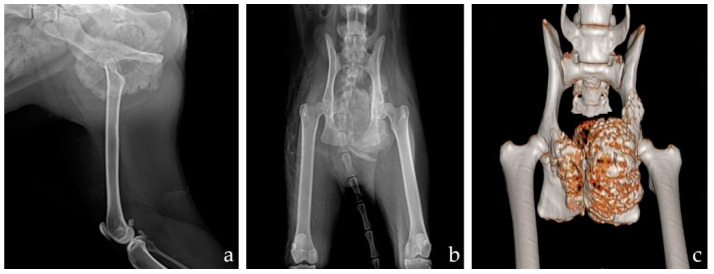
Pre-operative mediolateral radiographs of the left hind limb (**a**) and ventrodorsal pelvic radiographs (**b**). 3D reconstruction image after CT (**c**). There was a 4.5 × 3 × 5.4 cm mass expanding from the ischium to the ilium with a spiculated periosteal reaction.

A 5-year-old spayed female Turkish Angora cat weighing 3.4 kg was referred with a 1-month history of constipation and intermittent weight-bearing lameness in the left hindlimb. Upon physical examination, a mass was palpated in the pelvic cavity through rectal palpation. Abdominal radiographs revealed a spiculated periosteal reaction around the left acetabulum and expansion of the colon. In addition, the rectum was laterally oppressed and displaced by the mass in the pelvic cavity (Figure 1). Thoracic radiographs and blood examination revealed no remarkable findings. Computed tomography (CT) of the pelvis revealed a mass measuring 4.5 × 3 × 5.4 cm, which originated in the ischiatic table. However, there was no evidence of osteolysis in the pelvis and femur or metastasis on abdominal and thoracic CT. Pre-operative bone biopsy revealed predominant bone marrow, fragments of well-differentiated mature cortical bone, blood, and no malignant cells that would suggest an osteoma. Based on the results of these examinations, 3D-printed pelvic endoprosthesis and micro-total hip replacement (mTHR) were planned to reconstruct the pelvis and preserve hindlimb function.

### 2.2. Planning

A patient-specific surgical resection guide and an endoprosthesis were designed. CT (AlexionTM, Canon Medical Systems Corporation, Otawara, Japan) images were obtained with a slice thickness of 1 mm and operating parameters of 120 kV and 12 mA, and were processed using the digital imaging and communication in medicine (DICOM) file format. Segmentation and 3D model reconstruction in the design of the prosthesis were processed using a computer-aided design software program (Mimics; Materialize, Sheffield, United Kingdom).

**Figure 2:**
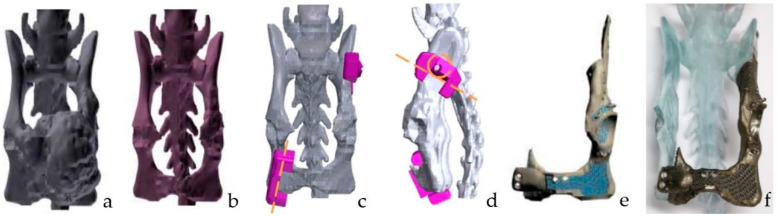
The pre-operative design process for hemipelvectomy. Segmentation and 3D reconstruction of the pelvis with the tumor were performed (**a**). Then, 3D modeling of the pelvis without the tumor was made (**b**). Design and manufacturing of the surgical resection guide and implant were performed to match the reconstructed pelvis without the tumor (**c**–**e**). Porous structures for soft tissue ingrowth, bone ingrowth, and cement interlocking were planned in the titanium implant (**e**). Screws for suturing, K-wiring, and bone screws for fixation were made on this implant. Rehearsal surgery with a bone and titanium implant was performed before the actual surgery (**f**).

The region to be resected to achieve an incomplete surgical margin (<2 cm) was identified using the computer-aided design software (Figure 2) [6]. Left mid-caudal partial hemipelvectomy with mTHR (Micro THR. BioMedtrix Inc., Boonton, NJ, USA) and right caudal partial hemipelvectomy were planned [6]. Two resection guides were made to designate an accurate osteotomy site and drill holes for the bone screw needed for the intended reduction.

The pelvic endoprosthesis has two extension arms attached with a screw to the contralateral ischium and the ipsilateral ilium (Figure 2). It has a porous structure for soft tissue ingrowth and cement to interlock the acetabulum and plate connection to the ipsilateral ilium and contralateral ischium. Holes for sutures, K-wire for temporary reduction, and screw holes were designed to augment bone fixation. Furthermore, it was designed so that there would be no difference in the weight-bearing alignment between pre- and post-operation.

### 2.3. Production of the Endoprosthesis

A bone model for rehearsal surgery and endoprosthesis was produced. For accurate surgical planning, rehearsal surgery was performed using a 3D-printed bone model (Figure 2). The bone models for rehearsal surgery were printed using a 3D printer (RS6000, Uniotech, Shanghai, China) based on a previously described protocol [19]. A 3D-printed pelvic endoprosthesis, with a porous structure for cement interlocking and ingrowth of the soft tissue and bone, was made with titanium using a 3D printer (SLM 125, SLM Solution, Lübeck, Germany). In addition, there were bone screw holes on the contact surface of the bone and suturing holes.

### 2.4. Surgical Technique

The operation sequence was hemipelvectomy, pelvis reconstruction with metal 3D-printed prosthesis, and mTHR. General anesthesia was induced prior to surgery. The patient was stabilized with fluid therapy and was premedicated with 0.1 mg/kg IV hydromorphone (Dilid inj. mg; Hana Pharm, Seongnam, Korea) and 0.2 mg/kg IV midazolam (Midazolam Inj; Bukwang Pharmaceutical Co., Seoul, Korea). General anesthesia was induced with 4 mg/kg IV propofol (Anepol Inj., Hana Pharm, Seongnam, Korea) and maintained with 2% isoflurane (Ifran; Hana Pharm, Seongnam, Korea) in 100% oxygen. For prophylaxis, 22 mg/kg IV cefazolin (Cefazolin Inj; Chong Kun Dang Healthcare, Seoul, Korea) was administered at 90 min intervals throughout the surgery. The surgical technique used in this case was based on a previous study [10]. A modified craniolateral approach was used for both hemipelvectomy and mTHR.

The skin incision extended from the iliac crest and ended just caudally and distally to the greater trochanter. Soft tissue attachments, such as origin and ligament insertion, were removed from the pelvis to acquire a sufficient window for bone resection. Left mid-to-caudal and right caudal partial hemipelvectomy and resection of the pelvic tumor were performed using the resection guide. During right caudal partial hemipelvectomy, right FHNO was performed because of the infiltration of the osteoma in the medial wall of the acetabulum, unlike the pre-operative plan in the 3D program. The bony defect after tumor resection was reconstructed using the pelvic endoprosthesis. In addition, a titanium mesh (NS-3MA-100-06, Jeil Medical Corporation, Seoul, Korea) was fixed between the metal implants and the bone for further fixation with the fascia of the surrounding muscle, due to the large defect after bone tumor excision. mTHR in the acetabulum cup (12 mm), and femoral stem (#3 with a 3.6-mm diameter tip and 46-mm length) insertion with polymethylmethacrylate (PMMA) cement (Surgical Simplex P, Howmedica Intl, Limerick, Ireland) were performed. The ligaments and surrounding muscles were sutured with polydioxanone (PDS II; Ethicon, Johnson & Johnson, New Jersey) to a similar site. The resected tumor was commissioned for histopathological examination and bacterial culture. After the surgery, the cat was hospitalized in the intensive care unit. Fluid therapy, antibiotic treatment, analgesia, and additional treatment were provided as indicated.

### 2.5. Outcomes, Complication, and Follow-Up

**Figure 3:**
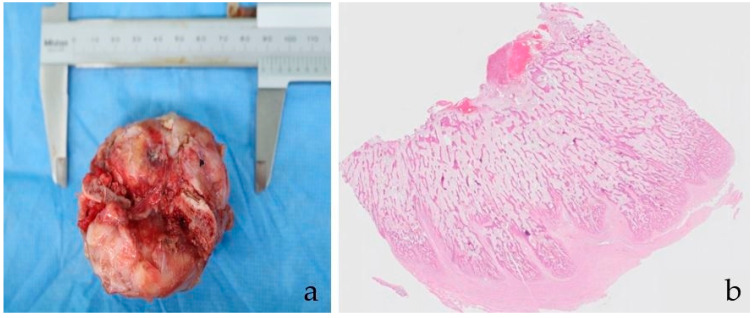
A 6.3 × 4 cm piece of the caudal part of the pelvis was removed (**a**). Post-operative biopsy specimen showed abundant proliferative new-bone formation (**b**). Trabeculae of immature bone were more densely packed superficially. There was no neoplasia within the examined tissue. Hematoxylin and eosin, ×0.5.

**Figure 4:**
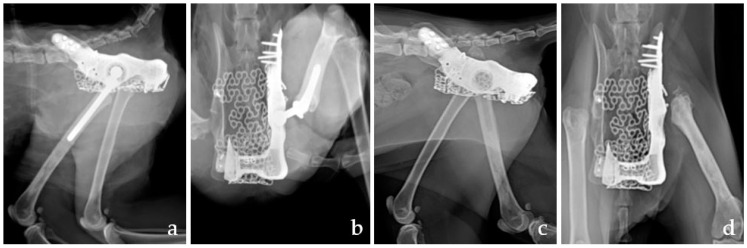
Post-operative mediolateral (**a**,**c**) and ventrodorsal radiographs (**b**,**d**). Immediate post-operative radiographs revealed the appropriate alignment of the pelvis and the position of the implant of mTHR (**a**,**b**). There was an osseointegration response between endoprostheses and bone in post-operative radiographs after 160 days (**c**,**d**). mTHR, micro total hip replacement.

The patient had delayed wound healing due to serosanguinous discharge after surgery. There was no infection, as confirmed by the bacterial culture of the exudate. Moreover, proprioceptive deficits and knuckling of the left hindlimb were consistent with sciatic neurapraxia, leading to femoral stem dislocation from the cup. Surgical debridement and femoral stem removal were performed 2 weeks after surgery. The delayed wound healing was successfully treated, and the sciatic neurapraxia gradually improved. The cat regained normal weight and defecation function 4 weeks post-operatively. The final histologic diagnosis was similar to that of the pre-operative interpretation (Figure 3). Local tumor recurrence did not develop until the last follow-up, 160 days after the surgery. Radiography at the final follow-up revealed the progression of osseointegration between the endoprosthesis and the bone (Figure 4). Further follow-up tests, including radiography, CT, and blood test, were refused by the owner because of the cost. However, the patient recovered with an almost-normal gait in physical examination, and the owners were satisfied with the functional outcome throughout the 2 years of telephone follow-ups. In particular, the owner was satisfied that the patient’s limb was spared and function was recovered.

## 3. Discussion

To the best of our knowledge, this is the first report to describe the total resection of an infiltrative osteoma in the pelvis of a cat, with the preservation of both hindlimbs, using a 3D resection guide and endoprosthesis. The patient had good long-term clinical outcomes. Although mTHR failed with neurapraxia, the patient showed normal gait through reconstruction using a 3D endoprosthesis; thus, its applicability in cats was confirmed.

The most-reported tumors in the pelvises of cats are osteosarcoma and chondrosarcoma, which are fast-growing and have a survival rate of less than 2000 days [2,6,9,20]. However, osteomas, composed of abnormally dense but otherwise normal bone formed in the periosteum, are rare in cats and arise mainly from the flat bones of the head. There has been no report of an osteoma in the pelvis of cats. An osteoma is a slow-glowing mass and generally does not cause clinical signs unless it interferes with adjacent structures [4,21]. Surgical resection is the primary treatment for bone tumors in cats. Amputation of the involved limb or excision of the involved bone can prolong survival more effectively [2,6,21]. In this case, a hemipelvectomy and THR for normal limb function was performed because an osteoma can progress into osteosarcoma. Lameness was also noted due to the size and location of the tumor, which led to problems in defecation.

Hemipelvectomy is the conventional treatment for patients with problems in the pelvic and proximal femur [6,18]. The pelvic bone is one of the weight-bearing axes that comprises of the acetabulum, iliac body, and sacroiliac joint. Aggressive resection of the hemipelvis is often necessary to achieve a tumor-free resection margin [6,9,10,13]. Any injury in this axis can disrupt the weight-bearing and gait system [7,15,16,18,22]. Limb length discrepancy and decreased acetabulum can impair walking function [23]. According to a previous report of 84 dogs and 16 cats who underwent hemipelvectomy, some owners reported that their dogs failed to adjust well to their amputee status and had limited mobility [9]. Although long-term satisfaction from the owner has been reported to be good, return to ambulation is typically delayed and requires rehabilitation. Prolonged post-operative analgesia and rehabilitation are important factors in the management of hemipelvectomy patients [6]. However, prosthetic reconstruction of the weight-bearing axis has the following advantages: early mobilization, long-term stabilization, and satisfactory function. It has been reported that the reconstruction of the weight-bearing axis with implants could prevent post-operative instability, leading to fractures [10,18,24]. Metal 3D-printing technology has the advantage of creating customized products similar to the pre-operative state of a bone without tumors. In this case, the implant was designed to fill the defect after the tumor removal and connect the bone to the implants firmly through bone proliferation. To regain the adduction and hamstring function of the hindlimbs following surgery, the origin of the muscles for this function must be reattached to the surrounding tissue. Moreover, the muscles were sutured to the 3D endoprosthesis and titanium mesh [8,11]. Although quality of life was not significantly affected, the reconstruction of these tendons and muscles is important to restore function [11,22]. The patient showed stabilization of the endo-prostheses through the progression of osseointegration and walking status without lameness.

An accurate resection of the hemipelvis through pre-operative assessment and planning is important for good outcomes, including sufficient surgical margins and survival times. Pre-operative examination involving CT, biopsy, and tumor staging to predict the duration of post-operative recovery and the degree of post-operative mobility is important for planning the surgery [6,11,22]. Hemipelvectomy traditionally necessitates the amputation of the ipsilateral hindlimb for the resection of the pelvic tumor if tumor invasion of the hindlimb is confirmed [7,9]. Modifications to hemipelvectomy were made during surgery, depending on the degree of tumor involvement [20]. A retrospective study showed a significant decrease in the local tumor recurrence rate with radical tumor excision (5 cm) when hemipelvectomy was performed in cats [24]. A wide margin resection with a normal tissue margin >2cm and fascial plane incorporation is required [6]. In this case, the accurate resection of the bone, sufficient surgical margin, and stable reduction of the bone and endoprosthesis were acquired through a resection guide. It has been reported that a resection guide can localize the surgical site accurately, eliminating the need for extra surgical time to restore the reduction between the implants and bone [13,15,17]. In addition, 3D modeling and printing offer significant advantages in assessing patient conditions. However, unlike the pre-operative plan in 3D software, it was confirmed that the bone tumor had invaded up to the medial wall of the right acetabulum; thus, right FHNO was performed. FHNO and resection of the acetabulum are needed for hindlimb preservation if the tumor is thought to extend cranially beyond the ischium [22]. It is unlikely that the tumor grew aggressively, or that an error in the process of producing guides and prostheses occurred, because there was no problem in the contact of the resection guide and endoprosthesis. It is possible that errors occurred in the process of identifying tumor invasion into the bone, including the transformation of the DICOM files and the identification of the tumor in the 3D program [25]. Moreover, CT should be acquired with a slice thickness of 0.5 mm or less to accurately specify the location and size of tumors. However, in this experiment, CT was measured in units of 1 mm [26]. Therefore, positron emission tomography–CT and magnetic resonance imaging, which are sensitive in detecting subtle lesions in the bone marrow, should also be considered in pre-surgical planning [11]. Although the excision was incomplete after surgery, histologically, the mass is composed of abundant proliferative new-bone formation, but no mitotic activity or additional criteria of malignancy are noted. There was no need for additional radiation therapy or chemotherapy based on post-operative histopathology [9]. Therefore, the patient remained stable, without local recurrence.

The most commonly reported post-operative complication after reconstruction with an endoprosthesis is infection [27]. Due to intraoperative hemorrhage, aggressive and extensive soft tissue dissection could contribute to post-operative edema, inflammation, and dehiscence [28,29]. In this case, the cat developed neuropraxia, leading to the dislocation of the stem out of the cup without evidence of infection. Protection of the sciatic nerve is essential for preserving post-operative function [30]. Although appropriate implant positioning and orientation were confirmed on immediate post-operative radiographs, excessive hip joint motion and knuckling due to sciatic neuropraxia may have been the cause of the stem dislocation in the femur when the patient walked and ran [31]. Therefore, the dislocated stem was removed, and the patient was successfully treated with surgical debridement. Hind limb function gradually improved and showed no lameness because the weight-bearing axis was maintained.

The limitations of our technique include the prolonged preparation and surgical time. In this case, it took a month to diagnose and operate. These surgical methods need to be avoided if a malignant tumor requires immediate surgery due to severe clinical symptoms. Therefore, it is necessary to examine the prognosis and complications of such surgeries for malignant tumors.

## 4. Conclusions

The surgical excision of osteoma is simple and could be curative without recurrence. Early treatment could be recommended for cats with osteoma, but aggressive surgical excision can result in an unacceptable quality of life. The use of 3D-printed pelvic prostheses for reconstructing bony defects after resection is safe and does not cause additional complications. Although there were complications, such as neuropraxia and dislocation of the stem of THR associated with THR, and minor complications such as delayed wound healing with serosanguinous discharge, they all healed well. Therefore, these techniques can provide satisfactory results for both patients and cats in terms of the endoprosthesis and the limb’s function. Although this treatment concept has already been applied in dogs, it is worth reporting in cats.

## Data Availability

Not applicable.
